# Antimycobacterial Activity of Constituents from *Foeniculum vulgare* var. Dulce Grown in Mexico

**DOI:** 10.3390/molecules17078471

**Published:** 2012-07-13

**Authors:** Patricia C. Esquivel-Ferriño, Juan Manuel J. Favela-Hernández, Elvira Garza-González, Noemí Waksman, María Yolanda Ríos, María del Rayo Camacho-Corona

**Affiliations:** 1 Facultad de Ciencias Químicas, Universidad Autónoma de Nuevo León. Av. Universidad s/n, Ciudad Universitaria, San Nicolás de los Garza, Nuevo León, C.P. 66451, Mexico; 2 Facultad de Medicina, Universidad Autónoma de Nuevo León. Madero y Aguirre Pequeño, Mitras Centro, Monterrey, Nuevo León, C.P. 64460, Mexico; 3 Centro de Investigaciones Químicas, Universidad Autónoma del Estado de Morelos. Av. Universidad 1001, Col. Chamilpa, Cuernavaca, Morelos, C.P. 62209, Mexico

**Keywords:** *Foeniculum vulgare (F. vulgare)*, *Mycobacterium tuberculosis*, multidrug resistant (MDR), 5-hydroxyfuranocoumarin

## Abstract

Bioassay guided fractionation of an antimycobacterial extract of *Foeniculum vulgare* var dulce (Apiaceae) led to the isolation and characterization of 5-hydroxyfurano-coumarin. The chemical structure of this compound was elucidated by ^1^H and ^13^C (1D and 2D) Nuclear Magnetic Resonance (NMR) spectroscopy. In addition, the active fractions were analyzed by GC-MS and seventy eight compounds were identified; the major compounds were 1,3-benzenediol, 1-methoxycyclohexene, *o*-cymene, sorbic acid, 2-hydroxy-3-methyl-2-cyclopenten-1-one, estragole, limonene-10-ol and 3-methyl-2-cyclopenten-1-one. Twenty compounds identified in the active fractions were tested against one sensitive and three MDR strains of *Mycobacterium tuberculosis* using the Alamar Blue microassay. Compounds that showed some degree of antimycobacterial activity against all strains tested were the following: linoleic acid (MIC 100 µg/mL), oleic acid (MIC 100 µg/mL), 1,3-benzenediol (MIC 100–200 µg/mL), undecanal (MIC 50–200 µg/mL), and 2,4-undecadienal (MIC 25–50 µg/mL), the last being the most active compound. To our knowledge, this is the first report of the presence of 5-hydroxy-furanocoumarin in *F. vulgare*.

## 1. Introduction

It is estimated that one third of the world’s population is infected with *Mycobacterium* *tuberculosis*. In 2010, there were 8.8 million new cases of tuberculosis (TB), 1.1 million deaths from TB among HIV-negative people and an additional 0.35 million deaths from HIV-associated TB. *M. tuberculosis* has developed resistance to first and second line anti-tuberculosis drugs [1], therefore, there is a need to develop new drugs to combat multi-drug resistant (MDR) and extremely drug resistant (XDR) *M. tuberculosis*, and continuous efforts are under way in the search for novel bioactive compounds to develop new anti-tuberculosis drugs. To this end, bioactive compounds of natural origin, particularly from plants, are gaining significance. 

*Foeniculum vulgare* Mill (Apiaceae), commonly known as fennel, is used as a spice and condiment in foods and cosmetics for its sweet flavor, aroma, and food preservation capability [2]. Fennel is native to Southern Europe and the Mediterranean region, but nowadays it is widely cultivated throughout the temperate and tropical regions of the World. Fennel was introduced in Mexico, where it is cultivated in home gardens and very often found free along roadsides and streams in the canyons of the Sierra Madre. It is used as a tea for stomach aches for its antispasmodic and carminative effects. In addition it promotes intestinal peristalsis and acts as an expectorant, and is associated with the diuretic action [3]. It is also recommended to cure various respiratory diseases [4].

Some chemical and biological studies have shown that fennel is an excellent source of antioxidants [5,6]. Fennel essential oil has a potent hepatoprotective action against CCl_4_-induced hepatic damage in rats, and D-limonene and β-myrcene were proposed to be the compounds potentially responsible for the activity [7]. Fennel oil was reported to exhibit estrogenic activity, promote menstruation, alleviate the symptoms of female climacterium, and increase libido [8]. Anethole, a component of fennel essential oil, inhibits TNF-induced cellular responses; TNF-mediated signaling is associated with both inflammation and carcinogenesis, thus fennel could be a chemopreventive agent [9]. Extracts and essential oils obtained from *F. vulgare* have shown antibacterial activity [10,11,12,13]. Dillapional and scopoletin were found to be the antimicrobial principles of fennel stems, which exhibited a potent antimicrobial activity against bacteria and fungi [14]. Our research group prepared a hexane extract from stems and leaves of *F. vulgare* var dulce grown in Mexico, this extract showed activity against one sensitive (MIC 200 µg/mL) and four monoresistant strains (MIC 100 µg/mL) of *M. tuberculosis* [15] using the Alamar Blue microassay. Thus, the aim of this study was to carry out the isolation and characterization of the antimycobacterial compounds from the hexane extract of *F. vulgare* var dulce grown in México.

## 2. Results and Discussion

### 2.1. Chemical Analysis

**Figure 1:**
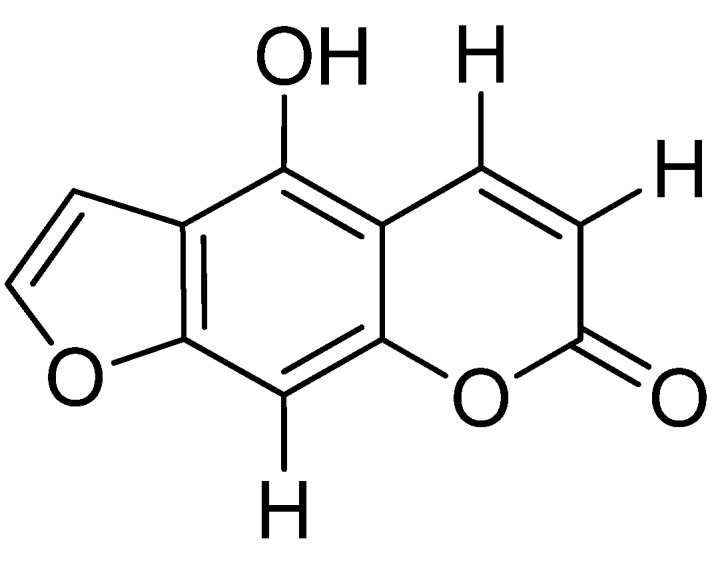
Chemical structure of 5-hydroxyfuranocoumarin.

The hexane extract of *F. vulgare* which showed anti-tuberculosis activity [15] was submitted to fractionation by column chromatography giving 10 fractions; all fractions were further tested against *M. tuberculosis* H37 Rv. The only fractions that showed activity were F-7 (MIC 50 µg/mL), F-8 (MIC 12.5 µg/mL), and F-9 (MIC 50 µg/mL). The other fractions did not show activity at concentrations lower or equal to 200 µg/mL. F-8 was the most abundant fraction (4.63 g) and thus was treated with several conventional chromatographic methods, which allowed the isolation of the major compound 5-hydroxyfuranocoumarin, known as bergaptol (**1**, Figure 1). This furanocoumarin is reported for the first time in fennel. The structure of this compound was established unequivocally through analysis of its ^1^H- and ^13^C-NMR spectroscopic data and by comparison of an authentic sample. It is important to mention that furanocoumarins have been found in fennel [14,16,17], and bergaptol could have antimycobacterial activity since it has been reported that some compounds of this type had activity against *M. tuberculosis* [18].

In addition, GC-MS analysis was carried out on F-7, F-8 and F-9 in order to determine their volatile constituents. Retention indices for all the compounds were determined according to the Kovats retention index method using C_8_-C_23_ n-alkanes as standards. The individual compounds were identified by their mass spectra fragmentation patterns using the NIST 1.7 library database, retention indices [19], and with authentic samples. From GC-MS analysis of F-7, 19 compounds were identified (Table 1), constituting 100% of the volatile constituents. These included two monoterpenes (1.53%), three sesquiterpenes (6.87%), four aromatics (33.80%), two aliphatic aldehydes (1.65%), two fatty acids (1.54%), and six aliphatic oxygenated compounds (54.61%). The major compounds identified in this fraction were 1,3-benzenediol (29.59%), 1-methoxycyclohexene (30.58%) and 2-hydroxy-3-methyl-2-cyclopenten-1-one (11.81%).

Twenty eight compounds were identified in F-8 (Table 2), constituting 100% of the volatile principles. These included nine monoterpenes (38.69%), seven sesquiterpenes (25.97%), five aromatics (21.56%), two aliphatic aldehydes (0.68%), and five aliphatic oxygenated compounds (13.10%). The major identified volatiles were *o*-cymene (19.23%), 1,3-benzenediol (14.91%), and limonene-10-ol (9.04%). Finally, from the F-9, 34 chemicals were identified (Table 3), constituting 100% of the volatile compounds. These included seven monoterpenes (10.75%), seven sesquiterpenes (14.55%), four aromatics (26.16%), four aliphatic aldehydes (4.05%), eleven oxygenated compounds (44.49%). The major compounds were sorbic acid (17.34%), 1,3-benzenediol (12.61%), estragole (10.49%), and 3-methyl-2-cyclopenten-1-one (7.70%).

**Table 1:** Chemical composition of *F. vulgare* (fraction 7).

Chemical compound	^a^ Retention time	Percentage	^b^ Retention Index
3-Hexene-2,5-diol	5.19	4.32	921
3-Methyl-2-cyclopenten-1-one	6.13	1.77	960
1,3-Benzenediol	9.21	29.59	1070
1-Methoxycyclohexene	10.93	30.58	1217
(1 *S*-(1α,3α,6α))-Bicyclo(4.1.0)hept-4-en-3-ol	12.60	0.59	1176
6-Hydroxy- *exo*-(±)-bicyclo(3.2.1)octan-3-one	13.35	2.71	1199
α-Thujone	13.68	0.94	1209
4-Ethyl-1,2-dimethylbenzene	13.84	1.30	1214
2-Hydroxy-3-methyl-2-cyclopenten-1-one	14.17	11.81	1224
Anethole	19.82	1.18	1398
Eugenol	22.45	1.73	1483
Bisabolene	23.40	1.58	1515
9-Acetoxynonaldehyde	24.26	0.73	1544
Carotol	25.73	3.73	1594
Cedrol	25.90	1.56	1600
2,14-Pentadecadienal	31.45	0.92	1804
Linoleic acid	32.12	0.50	1830
Oleic acid	34.53	1.04	1926
6,10,14-Trimethyl-2-pentadecanone	35.98	3.42	1986

^a^ GC, identification based on retention times of compounds on an SGE-BPX5 capillary column; MS, identified based on computer matching of mass spectra with NIST 1.7 library; ^b^ Retention index (RI) relative to *n-alkanes* on an SGE-BPX5 capillary column.

**Table 2:** Chemical composition of *F. vulgare* (fraction 8).

Chemical compound	^a^ Retention time	Percentage	^b^ Retention Index
3-Hexen-2-one	5.14	1.66	919
2-Ethyl-2-butenal	6.42	0.32	972
2,4-Dimethylpentanal	7.34	0.36	908
Camphene	8.31	1.38	1041
3-Hexene-2,5-dione	8.55	1.03	1049
3-Methyl-2-cyclopenten-1-one	8.67	4.29	1052
1,3- Benzenediol	9.17	14.91	1069
*o*-Cymene	10.87	19.23	1215
Terpinolene	12.56	0.15	1175
Isocitronellol	12.97	0.17	1187
2-Methyl-3-(1-methylethenyl)-,(1α,2α,3α)- cyclohexanol	13.11	0.06	1191
2-Hydroxy-3-methyl-2-cyclopenten-1-one	14.07	4.14	1320
1,2,4,5-Tetramethylbenzene	14.97	0.25	1248
Limonen-10-ol	16.32	9.04	1287
Fenchyl acetate	17.00	3.85	1310
Cyclamen aldehyde	21.78	1.38	1461
6,6,7-Trimethyl-3-octyne-2,5-dione	21.87	1.97	1464
Linalol isovalerate	21.99	2.48	1468
Ethyl linalool	22.08	3.16	1471
Bisaboleno	23.41	2.46	1515
Methylisovalerate	23.49	1.65	1517
Apofarnesol	23.60	4.69	1521
Kessane	23.85	6.31	1530
Thymohydroquinone	24.60	2.06	1555
Germacrene D-4-ol	25.11	3.50	1575
Viridiflorol	25.69	4.05	1592
Cedrol	25.92	2.48	1600
Isoeugenol acetate	26.29	2.96	1614

^a^ GC, identification based on retention times of compounds on an SGE-BPX5 capillary column; MS, identified based on computer matching of mass spectra with NIST 1.7 library; ^b^ Retention index (RI) relative to *n-alkanes* on an SGE-BPX5 capillary column.

**Table 3:** Chemical Composition of *F. vulgare* (fraction 9).

Chemical compound	^a^ Retention time	Percentage	^b^ Retention Index
3-Hexene-2,5-diol	5.19	1.44	921
2,3-Dimethyl-2,3-butanediol (pinacol)	5.77	3.76	945
3-Methyl-cyclohexanol	6.12	1.32	960
2-Methyl-2-pentenal	6.42	0.69	972
2,2-Dimethyl-3-propyl-oxyrane	7.05	2.77	979
Heptadienol	7.56	4.53	1016
4,4-Dimethylpent-2-enal	8.31	1.92	1041
3-Hexen-2,5-dione	8.55	0.73	1049
3-Methyl-2-cyclopenten-1-one	8.67	7.70	1052
1,3-Benzenediol	9.17	12.61	1069
α-Pinene oxide	10.07	1.36	1099
Sorbic acid	10.87	17.34	1123
γ-Terpinene	11.74	0.99	1150
Fenchone	12.73	0.43	1180
Citronellol epoxide	13.38	0.62	1200
Fenchyl acetate	14.10	5.04	1220
3,3,6-Trimethyl-1,5-heptadien-4-one	15.27	0.43	1257
Estragole	16.31	10.49	1288
Methyl chavicol	16.99	2.07	1309
Carvone	18.36	1.54	1451
2,4-Undecadienal	18.67	1.14	1460
*p*-Anisaldehyde	18.78	0.99	1463
Undecanal	20.15	0.30	1408
3-Hydroxydodecanoic acid	21.86	0.78	1464
Caryophyllene	21.99	1.15	1466
Methyl isovalerate	23.49	1.17	1517
Tridecanol	25.10	2.31	1571
Germacrene D-4-ol	25.25	1.17	1575
Globulol	25.69	2.65	1590
Trans-β-elemenone	25.91	4.31	1602
Β-Himachelene oxide	26.29	2.69	1614
Cedrene-3-one	27.17	1.15	1645
β-Eudesmol	27.34	1.03	1651
Sedanenolide	29.25	1.38	1720

^a^ GC, identification based on retention times of compounds on an SGE-BPX5 capillary column; MS, identified based on computer matching of mass spectra with NIST 1.7 library; ^b^ Retention index (RI) relative to *n-alkanes* on an SGE-BPX5 capillary column.

Recently, the chemical composition of hexane extract obtained from dried leaves of *F. vulgare* var. *azoricum* (Mill) from Turkey was studied by GC-MS [12]. Monoterpenes, sesquiterpenes, aromatics, fatty acids and others were identified in the whole extract. Though our study only reports the chemical composition of active fractions the above study was in agreement with ours because we found a similar composition. In contrast, we found aldehydes and other oxygenated compounds in the active fractions. Aldehydes such as undecanal, dodecanal, and 2-dodecenal had been reported in the hexane fraction of seeds of *F. vulgare* [20]. With respect to the variation of chemical composition of the other oxygenated compounds found in the active fraction, it can be explained by genotypic and environmental differences between the studied species.

### 2.2. Antimycobacterial Activity

Twenty compounds identified in the active fractions were purchased and tested first against *M. tuberculosis* H37Rv. The results (Table 4) show that eugenol, oleic acid, linoleic acid, and 1,3-benzenediol had MIC values in the range of 100 to 200 μg/mL, thus contributing to the antimycobacterial activity of F-7.

**Table 4:** Antimycobacterial activity of some compounds from *F*. *vulgare*.

	*M. tuberculosis* MIC (µg/mL)			
Fraction/Chemical compound	H37 Rv	M-10	M-15	M-26
**Fraction 7**				
α-Thujone	>200	ND	ND	ND
Anethole	>200	ND	ND	ND
Eugenol	100	ND	ND	ND
Oleic acid	100	100	100	100
Linoleic Acid	100	100	100	100
2-Hydroxy-1-methyl-1-cyclopenten-3-one	>200	ND	ND	ND
1,3-Benzenediol ^a^	200	200	100	100
**Fraction 8**				
Terpinolene	200	ND	ND	ND
*o*-Cymene	50–100	ND	ND	ND
3-Methyl-2-cyclopenten-1-one	>200	ND	ND	ND
Isocitronellol	>200	ND	ND	ND
**Fraction 9**				
Fenchylacetate	>200	ND	ND	ND
Carvone	>200	ND	ND	ND
*p*-Anisaldehyde	100	ND	ND	ND
Fenchone	200	ND	ND	ND
Methylchavicol	>200	ND	ND	ND
Estragole	100–200	ND	ND	ND
Undecanal	100	200	100	50
2,4-Undecadienal	25	50	25	25
2,3-Dimethyl-2,3-butanediol	>200	ND	ND	ND
Ethambutol	2.0	15	15	15

H37Rv: Sensitive to izoniazid, rifampicin, ethambutol and pyrizanamide. M10, M15, M26: multidrug-resistant. ND: not determined; ^a^ 1,3-Benzenediol was found in fractions 7, 8 and 9.

On the other hand, the following compounds gave antimycobacterial activity in F-8: terpinolene, *o*-cymene, and 1,3-benzenediol having MIC values in the range of 50 to 200 μg/mL. Compounds in F-9 such as *p*-anisaldehyde, fenchone, estragole, undecanal, and 2,4-undecadienal also had antimycobacterial activity (MIC 25 to 200 μg/mL). Oleic acid, linoleic acid, 1,3-benzenediol, undecanal and 2,4-undecadienal were tested further against three MDR *M. tuberculosis* strains (Table 4).

From results we saw that the most active compound against the sensitive and MDR *M. tuberculosis* was 2,4-undecadienal, which was 12.5 times less active than the positive control ethambutol. It has been reported that aldehydes from *Coriandrum sativum* (Apiaceae) display antimicrobial activity, probably due their reactivity against nucleophilic groups (amino, hydroxy, sulfhydryl) in proteins associated with the cell membrane [21]. In another study, it was observed that the chain length of unsaturated aldehydes plays an important role in their antimicrobial activity, since they can behave as nonionic surfactants [22]. Thus, it is probable that the reactive ability of aldehydes groups plus the detergent-like properties make 2,4-undecadienal and undecanal active against *M. tuberculosis*. It is important to point out that our findings showed that the unsaturated aldehyde, undecanal showed weak antimycobacterial activity, while the α,β-unsaturated aldehyde 2,4-undecadienal was four times more active.

Apart from 2,4-undecadienal, the tested compounds that showed activity towards *M. tuberculosis* had MIC values higher than those found in the active fractions thus they may act in a synergistic way in the active fractions. On the other hand, it is important to mention that it is possible that one or more compounds identified in the active fractions that we did not have the chance to test could have antimycobacterial activity.

In the case of oleic acid and linoleic acid, it has been reported since 1956 that these acids affect the integrity of the cellular wall in the mycobacteria [23]. For the above effect to happen, the concentration of these fatty acids has to be considerably higher, compared to the concentration found in the OADC (0.6 µg/mL; PML Microbiological, Inc, Franklin Lakes, NJ, USA) used as enrichment medium to grow the mycobacteria. It is known that the majority of fatty acids are more effective against Gram positive than Gram negative bacteria. These facts suggest that the mechanism of their bactericidal action is due to a balance between the hydrophilic and hydrophobic portions of the molecule. The answer to why Gram positive bacteria are more sensitive than Gram negative bacteria to lipophilic agents it is due to the complexity of their cell walls [24]. In this sense it is important to mention that *M. tuberculosis* is considered to be a Gram positive bacterium with the additional presence of mycolic acids in its cell wall. Thus, the above could explain the activity of fatty acids on *M. tuberculosis*. Unsaturated long-chain fatty acids (oleic acid and linoleic acid) were found to be lethal for *M. bovis* and *M. tuberculosis* H37Ra. The inhibition of the function of enzymes associated with the cytoplasmic membrane suggested that killing was due to a detergent-like effect on this organelle [25].

On the other hand it was reported that antibacterial effect of long-chain unsaturated fatty acids such as linoleic acid, palmitoleic acid, oleic acid, and arachidonic acid was due to their inhibition of the bacterial enoyl-acyl carrier protein reductase (Fab I), an important enzyme involved in synthesis of fatty acids biosynthesis [26]. Thus, it is possible that the mechanism of inhibition could interfere in the synthesis of mycolic acids present in the cell wall of mycobacteria [27].

It is important to point out the presence of 1,3-benzenediol as one of the major compounds in the three active fractions. This compound had antimycobacterial activity against sensitive and MDR resistant strains tested; recently it has been reported that analogues of resorcinol showed activity against *M.tuberculosis* H37Rv with MIC values in the range of 2.5–60 µg/mL [28]. Although, the MIC values of resorcinol are higher we tested this compound against MDR clinical isolates strains obtaining positive outcomes. It is important to mention that other related phenolic compounds have demonstrated antimycobacterial activity *in vitro* and *in vivo* [29,30].

## 3. Experimental

### 3.1. Plant Material

Stems and leaves of *F. vulgare* var. dulce were collected in Pesquería, Nuevo León (Mexico) in July 2007. Plant material was provided and identified by Biologist Mauricio Gonzalez Ferrara; a sample with a voucher number 024771 was deposited at the Herbarium of the Department of Botany, Universidad Autónoma of Nuevo León, Mexico.

### 3.2. Chemical and Reagents

Hexane and ethyl acetate were of analytical grade and purchased from CTR (Monterrey, N.L. Mexico); fenchylacetate, α-tujone, carvone, *o*-cymene, *p*-anisaldehyde, fenchone, terpinolene, methylchavicol, *trans*-anethol, estragole, eugenol, 1,3-benzenediol, 2,3-dimethyl-2,3-butanediol (pinacol), 3-methyl-2-cyclopenten-1-one, undecanal, 2-hydroxy-1-methyl-1-cyclopenten-3-ona, isocitronellol, 2-4 undecadienal, oleic acid and linoleic acid, C_8-_C_23_ *n*-alkane standards, ceric sluphate, glycerol, dimethyl sulfoxide and Tween 80 were purchased from Sigma-Aldrich (St. Louis, MO, USA). Silica gel 60 size 0.040–0.063 mm (230–400 mesh) EMD (Darnstadt, Germany) was used for column chromatography. Thin-layer chromatography was performed on pre-coated aluminium sheets 20 × 20 cm (silica gel 60 F_254_, 0.2 mm, Merck, Darnstadt, Germany); Preparative Thin Layer Chromatography was performed on silica gel 60 F_254_ plates 0.5 mm, 20 × 20 cm (Merck). Biological assays: Middlebrook 7H9 broth was purchased from Difco Laboratories (Detroit, MI, USA); oleic acid/albumin/dextrose/catalase (OADC) was obtained from BBL/Becton Dickinson (Franklin Lakes, NJ, USA); Alamar Blue solution was obtained from Trek Diagnostics, (Westlake, OH, USA).

### 3.3. Equipment Utilized

1-D and 2-D ^1^H- and ^13^C-Nuclear Magnetic Resonance (NMR) experiments were performed on a Bruker 400 MHz spectrometer. Chemical shifts (ppm) were determined with TMS as internal standard. CDCl_3_ was used as a solvent. Gas Chromatography-Mass Spectra (GC-MS) analysis of active fractions was performed using a HP Agilent Technologies 6890 gas chromatograph equipped with a MSD 5973 quadrupolar mass detector in electron impact mode at 70 eV; Volatile compounds were separated on a BPX5 MS capillary column (25 m × 0.2 mm i.d., film thickness 0.3 µm). The oven temperature was set at 60 °C for 2 min, was then programmed at 4 °C /min to 200 °C and maintained for 10 min. Mass detector conditions were as follows: interface temperature, 200 °C; mass acquisition range 10–300. The carrier gas was helium at a flow rate of 1 mL/minute. Temperature of injector and the detector were set to 250 °C and 280 °C respectively. A standard solution of C_8_-C_23_-alkanes was used to obtain the index retention times. Identification of the volatile components was performed comparing their mass spectra with those of the NIST 1.7 library. Compound identification was verified based on relative index retention time [19] and mass spectra of authentic standards from Sigma-Aldrich. Semiquantitative data were calculated from the GC peak areas without using correction factors and were expressed as relative percentage (peak area %) of the total volatile constituent identified.

### 3.4. Extract Preparation and Isolation of 5-Hydroxyfuranocoumarin *(1)*

Powdered air-dried leaves and stems (944 g) were macerated with 18 L of hexane. The hexane was evaporated *in vacuo* to yield 7.72 g of crude extract; this extract was tested for antimycobacterial activity. The active extract was further fractionated by column chromatography on silica gel and eluted with a hexane/ethyl acetate gradient (F1 100:0; F2 98:2; F3 95:5; F4 95:5; F5 90:10; F6 90:10; F7 85:15; F8 80:20;70:30; F9 60;40; F10 50:50), yielding 10 fractions. Each fraction was analyzed by silica gel thin-layer chromatography; spots were visualized under UV light (254 and 365 nm) and sprayed with ceric sulphate. Each fraction was next tested against *M. tuberculosis* H37Rv using an Alamar Blue microassay. The only fractions that showed activity were F-7, F-8 and F-9. The most abundant fraction F-8 (4.63 g) was subjected to silica gel column chromatography and eluted with a hexane/ethyl acetate gradient (sf1 100:0; sf2 90:10; sf3 80:20; sf4 70:30; sf5 60:40; sf6 50:50) yielding six sub-fractions. Sub-fraction 2 (173.5 mg) was further chromatographed on silica gel using a hexane/ethyl acetate gradient (sf1 100:0; sf2 98:2; sf3 95:5; sf4 90:10; sf5 85:15) giving five sub-fractions. Sub-fractions 4 and 5 were pooled (80.2 mg) and purified by silica gel preparative chromatography eluted with hexane/ethyl acetate (90:10) affording compound **1** (12.9 mg, which corresponded to 0.00136% of the dried plant material) which was analyzed by NMR showing the following spectral data: ^1^H-NMR δ: 7.96 (1H, *s,* H-8), 7.38 (1 H, *d*, *J =* 5.6 Hz, H-4), 6.95 (*d*, *J* *=* 1.2 Hz, H-2′), 6.04 (1H, *d*, *J* = 5.6 Hz , H-3), 5.90 (1H,*d*, *J* *=* *1*.2 Hz, H-3′); ^13^C-NMR δ: 166.55 (C-2), 158.9 (C-7), 156.63 (C-8a), 146.15 (C-5), 142 (C-2′), 132.81 (C-3), 132.81 (C-6), 131.57 (C-4a), 131.57 (C-3′), 122.09 (C-8), 128.79 (C-4). The COSY spectrum exhibited two correlations (H-4/H-5 and H-2′/H-3′). From spectral analysis and comparison with an authentic sample, compound **1** was characterized as 5-hydroxyfuranocoumarin (Figure 1), which is reported for the first time in fennel.

### 3.5. Microorganisms

*M. tuberculosis* H37Rv ATCC 27294 sensitive to isoniazid, rifampicin, ethambutol and pyrizanamide was purchased in American type culture. The three multidrug resistant (MDR) clinical isolates were collected from sputum specimens of tuberculosis patients; these were kindly provided by Virgilio Bocanegra-García. 

### 3.6. Antimycobacterial Assay

Mycobacteria was cultured in Middlebrook 7H9 broth supplemented with 0.2% glycerol and 10% OADC at 37 °C for two weeks in order to reach logarithmic phase growth. The testing inoculum was prepared by diluting the bacterial suspension with culture medium to adjust turbidity to McFarland’s nephelometer No. 1 standard and it was then further diluted to 1:20 with same culture medium. Antimycobacterial activity was determined as previously described in the literature [15]. Briefly, in a 96-well sterile microtiter plate serial two-fold dilutions of each sample were prepared to a final volume of 100 μL. Then 100 μL *M. tuberculosis* testing inoculum was added to testing and control wells. The microtiter plate was incubated at 37 °C in a sealed plastic bag during five days. After incubation time, all the wells received the mixture of 20 µL Alamar blue and 12 µL 10% Tween 80. The plates were re-incubated at 37 °C for 24 h. Wells with a well-defined pink color were scored as positive growth, those wells with blue color were scored as inhibition of growth. Minimum Inhibitory Concentration (MIC) value was expressed as the lowest concentration of compound that caused 100% inhibition of mycobacterium growth. All assays were run in triplicate and ethambutol was used as positive controls. The samples were tested in the concentration range of 200 to 6.25 µg/mL.

## 4. Conclusions

The present study supports the fact that hexane extract of *F. vulgare var.* dulce offers an excellent opportunity to find active molecules against multidrug resistant *M. tuberculosis*. 2,4-Undecadienal was the most active compound found in this study. The dietary intake of fennel may lower the risk of *M. tuberculosis* infection, however, it is necessary to do *in vivo* studies to prove the above. This is also the first report of the presence of 5-hydroxyfuranocoumarin (**1**) in *F. vulgare*.
